# Midwifery and nursing students’ occupational health hazards and safety competence perceptions: a cross-sectional study from Türkiye

**DOI:** 10.1590/1980-220X-REEUSP-2025-0085en

**Published:** 2026-05-15

**Authors:** Aytül Hadımlı, Nurten Alan, İlkay Ünal, Nazan Tuna Oran

**Affiliations:** 1 Ege University, Faculty of Health Sciences, Department of Midwifery, Izmir,Türkiye.; 2 Dokuz Eylül University, Faculty of Nursing, Izmir, Türkiye.

**Keywords:** Occupational health, Nursing, Midwifery, Students, Saúde Ocupacional, Enfermagem, Tocologia, Estudantes

## Abstract

**Objective:**

The study was aimed at investigating occupational health hazards that midwifery and nursing students are exposed to during their clinical practice and their perceptions of occupational safety competence.

**Method:**

The study included 289 midwifery and 458 nursing students. Data were collected using the Student Information Form and Occupational Health and Safety Perceived Competency Scale.

**Results:**

During their clinical practice, students were most frequently exposed to sharps injuries. Most students who experienced an occupational accident reported the incident to more than one authority. Multiple linear regression analysis indicated that the factors predicting occupational safety competence perceptions were department, choosing the department willingly, perceiving the training adequate, and being aware of the occupational health and safety measures.

**Conclusion:**

This study identified that midwifery and nursing students are most frequently exposed to sharps injuries during clinical practice, primarily due to inexperience and inattention. Students who received adequate occupational health and safety training, chose their department willingly, and were aware of preventive measures demonstrated higher perceived occupational safety competence. The findings confirm that both educational background and personal factors significantly affect students’ safety perceptions, highlighting the need for comprehensive and standardized occupational health and safety training in health education programs.

## RESUMO

**Objetivo:**

O estudo teve como objetivo investigar os riscos ocupacionais à saúde aos quais estudantes de obstetrícia e enfermagem estão expostos durante a prática clínica e suas percepções de competência em segurança ocupacional.

**Método:**

O estudo incluiu 289 estudantes de obstetrícia e 458 estudantes de enfermagem. Os dados foram coletados por meio do Formulário de Informações do Estudante e da Escala de Competência Percebida em Saúde e Segurança Ocupacional.

**Resultados:**

Durante a prática clínica, os estudantes foram mais frequentemente expostos a lesões por perfurocortantes. A maioria dos estudantes que sofreu um acidente ocupacional relatou o incidente a mais de uma autoridade. A análise de regressão linear múltipla indicou que os fatores preditores das percepções de competência em segurança ocupacional foram o curso frequentado, a escolha voluntária do curso, a percepção de adequação do treinamento e a conscientização sobre as medidas de saúde e segurança ocupacional.

**Conclusão:**

Este estudo identificou que estudantes de obstetrícia e enfermagem são mais frequentemente expostos a lesões por perfurocortantes durante a prática clínica, principalmente devido à inexperiência e desatenção. Estudantes que receberam treinamento adequado em saúde e segurança ocupacional, escolheram seu curso voluntariamente e estavam conscientes das medidas preventivas apresentaram maior competência percebida em segurança ocupacional. Os achados confirmam que tanto a formação educacional quanto fatores pessoais afetam significativamente as percepções de segurança dos estudantes, destacando a necessidade de treinamento abrangente e padronizado em saúde e segurança ocupacional nos programas de educação em saúde.

## INTRODUCTION

The International Labour Organization (ILO) defines occupational health and safety as the prevention of work-related injuries and diseases and the protection and promotion of workers’ health. It also aims to improve working conditions and the work environment. The ILO reports that approximately 2.3 million workers worldwide are affected by work-related accidents or diseases each year. In Türkiye, despite the precautions taken, the prevalence of occupational accidents remains high. According to 2016 ILO data, the rate of non-fatal occupational injuries per 100,000 workers in Türkiye was 1,530, while the fatality rate was 7.5^(1)^. In Türkiye, Law No. 6331 has legally shaped both health and safety conditions for employees and their occupational health and safety training. According to Article 7/3 of this law, “It is the employer’s responsibility to provide training and practical training for both apprentices and interns.” However, apprentices and students who are undertaking internships in institutions must also receive occupational health and safety training. According to the law, occupational health and safety courses completed by apprentices and interns at their educational institutions may substitute for basic workplace training if approved by the employer^(2)^.

Employees may encounter many dangers depending on the nature of their work. The healthcare sector carries a higher risk of occupational accidents and diseases compared to many other sectors. Hospitals are classified as very hazardous workplaces according to the “Workplace Hazard Classes Communiqué”^(3,4,5)^. This sector, which poses risks for healthcare workers, also presents risk for students in health disciplines who are preparing to enter this field^(6)^. Studies indicate that many hospital accidents involve students, with approximately half experiencing at least one accident during internships, most commonly sharps injuries^(4,5,7,8,9,10,11)^. Nursing and midwifery students, who constitute one of the largest groups in health education, face numerous occupational hazards during clinical practice. Their limited experience and knowledge may increase their risk of workplace accidents and occupational illnesses. Therefore, equipping them with adequate knowledge and awareness of occupational health and safety is essential. This study aims to examine the occupational health hazards faced by midwifery and nursing students during clinical practice and their perceptions of occupational safety competence.

## METHOD

### Design of Study

This was a cross-sectional study.

### Sample Definition

The study was conducted at two universities in the Aegean region of Türkiye between October 2022 and December 2022. These universities were selected because they established midwifery and nursing programs with large student populations and accessible clinical practice networks. Students were invited to participate at the beginning of the fall semester in October 2022, and data collection continued until December 2022.

Nursing students from one of the universities and midwifery students from the other university were included in the study. All second-, third-, and fourth-year students who had completed at least one term of clinical practice were considered eligible for inclusion. As part of their undergraduate curriculum, these students had already received formal theoretical instruction in infection control, patient and workplace safety, and clinical procedures before beginning their internships. Students who had not yet started clinical practice or who declined to provide informed consent were excluded from the study. Among the eligible students, 370 were from the Midwifery Department and 790 were from the Nursing Department. Of these, 289 midwifery students and 458 nursing students agreed to participate. Therefore, the study was conducted with 747 students. Recruitment was carried out through in-class announcements by instructors and institutional email invitations. Participation was voluntary, and written informed consent was obtained from all participants.

Participation rates were calculated separately for each department. In the Midwifery Department, 289 of 370 eligible students participated, yielding a participation rate of 78.1%. In the Nursing Department, 458 out of 790 eligible students participated, resulting in a participation rate of 58.0%.

The adequacy of the sample size of 747 students was evaluated through a post-hoc power analysis based on a 95% confidence level, a 5% significance level (α = 0.05), and a medium effect size (Cohen’s d = 0.5). The analysis indicated that the statistical power of the study exceeded 95%, demonstrating that the sample size was sufficient for statistical analyses.

### Data Collection

*Student information form:* The form, prepared in line with the literature, consists of 33 items^(5,9)^. Of these items, 12 assess students’ sociodemographic characteristics, and 11 assess their knowledge and experience related to occupational health and safety. The Student Information Form consists of two sections: (a) 12 questions regarding sociodemographic characteristics (e.g., age, gender, department, year of study, income status, and chronic illness); and (b) 11 questions assessing knowledge and experience related to occupational health and safety, including prior training, awareness of protective measures, history of occupational accidents, and reporting behavior.

*Occupational Health and Safety Perceived Competency Scale:* The scale, developed by Kocaay and Ocaktan to assess employees’ perceptions of occupational health and safety competence, consists of 29 items. Responses to the items are rated on a five-point Likert-type scale ranging from 1 to 5 (1 = not at all, 2 = a little, 3 = partly, 4 = well, and 5 = very well). No items are reverse scored. The minimum and maximum possible scores that can be obtained from the scale are 29 and 145, respectively. The Cronbach’s alpha reliability coefficient of the scale was 0.91 in the original study by Kocaay and Ocaktan^(12)^, and 0.97 in the present study, indicating excellent internal consistency.

### Data Analysis

The data obtained from the study were analyzed using SPSS 25.0. The normality of the data distribution was assessed using the Kolmogorov-Smirnov test. Since the data were normally distributed, parametric tests were applied. To compare the mean scores of the Occupational Health and Safety Perceived Competency Scale across independent variables, the independent samples t-test was used for comparisons between two groups, while one-way analysis of variance (ANOVA), Tukey Post hoc HSD analysis, and multiple linear regression analysis were used for comparisons involving more than two groups. A P-value of less than 0.05 was considered statistically significant.

### Ethical Aspects

This study was approved by the XXX University Scientific Research and Publication Ethics Committee (Protocol No: 09/12-1580). The participating students were informed about the purpose and scope of the study, and written informed consent was obtained from all participants.

## RESULTS

The mean age of the students participating in the study was 21.10 ± 1.50 (min: 19 max: 33) years. Of the participants, 76.6% were female, 61.3% were nursing students, and 38.7% were midwifery students. Additionally, 55.4% lived in dormitories, 53.1% reported having an income equal to their expenses, 90.9% did not work in a paid job, 79.5% had health insurance, and 93.0% did not have a chronic disease (Table 1).

**Table 1:** Sociodemographic and health-related characteristics of the participating students – Izmir, Türkiye, 2022.

Characteristics	Number	%
**Mean age:** 21.10 ± 1.50 (min: 19, max: 33) years		
**Sex**		
Female	572	76.6
Male	175	23.4
**Department**		
Nursing	458	61.3
Midwifery	289	38.7
**Year at school**		
2	254	34.0
3	207	27.7
4	286	38.3
**Place of residence**		
Home shared with friends	166	22.2
Home with the family	167	22.4
Dormitory	414	55.4
**Income status**		
Income more than expenses	85	11.4
Income equal to expenses	397	53.1
Income less than expenses	265	35.5
**Employment status**		
Employed	68	9.1
Not employed	679	90.9
**Health insurance**		
Yes	594	79.5
No	153	20.5
**Presence of an illness for which he or she is constantly treated**		
Yes	52	7.0
No	695	93.0
**Type of the illness (n:52)**		
Endocrine system	14	26.9
Immune system	9	17.3
Gastrointestinal system	8	15.4
Nervous system	6	11.5
Cardiovascular system	5	9.6
Respiratory system	5	9.6
Psychiatric	5	9.6
**Total**	**747**	**100**

The majority of the participants were female nursing students in their second or fourth year, most of whom resided in dormitories. Most had health insurance, were not employed, and did not have any chronic illness.

Most students reported receiving occupational health and safety training within applied courses and considered this training adequate. Basic protective measures, such as hand hygiene, glove use, and mask use, were frequently practiced during clinical training.

Students received occupational health and safety training in various settings, most commonly as part of practical courses. A total of 76.3% stated that the training they received was sufficient. During clinical practice, the most frequently used protective measures were hand hygiene, gloves and masks (Table 2).

**Table 2:** Findings regarding the students’ occupational health and safety education – Izmir, Türkiye, 2022.

Characteristics	Number	%
**The place where they receive occupational health training^†^**		
Occupational health and safety course at school	295	39.5
Within the Scope of the applied courses	501	67.1
At the institution where the participant practice	54	7.2
In a private course that provides occupational health education	79	10.6
**Considering the occupational health and safety training received as adequate**		
Yes	570	76.3
No	177	23.7
**Being aware of the measures to be taken to prevent occupational accidents**		
Yes	668	89.4
No	79	10.6
**Having a regular health screening before clinical practice**		
Yes	337	45.1
No	410	54.9
**Protective measures taken during clinical practice^†^**		
None	7	0.9
Hand washing	684	91.6
Wearing gloves	675	90.4
Wearing a face mask	645	86.3
Wearing glasses/visors	41	5.5
Wearing an apron	278	37.2
**Total**	**747**	**100**

^†^Multiple answers.

The most common occupational accident was sharps injuries, primarily caused by inattention or lack of experience. Most incidents were reported, typically to the training nurse or relevant institutional units.

Among the students, 87.6% reported not experiencing any occupational accident during their clinical practice. The most common type of accident was sharps injuries, and the most frequently reported cause was carelessness. Students who experienced an occupational accident reported the incident to more than one authority. The training nurse was the authority to whom incidents were most commonly reported (Table 3).

**Table 3:** Findings about occupational accidents the participating students had – Izmir, Türkiye, 2022.

Characteristics	Number	%
**Having had an occupational accident**		
Yes	93	12.4
No	654	87.6
**Type of the occupational accident^‡^**		
Sharps injuries	77	10.8
Contamination with blood or body fluids of the patient	14	1.9
Falls	9	1.2
Exposure to physical/verbal violence	4	0.5
Exposure to X-Ray	1	0.1
Chemical injury	1	0.1
**Cause of the accident^‡^**		
Inattention	59	7.9
Lack of practice	33	4.4
Lack of personal protective equipment	13	1.7
Fatigue, sleeplessness	17	2.3
Stress, anxiety	12	1.6
**Authority to whom the accident is reported^‡^**		
The nurse giving training	37	5.0
Hospital employee health and safety unit	32	4.3
Faculty/internship-application supervisor	30	4.0
The infection control unit	17	2.3
No reporting	28	3.7

^‡^Students who had an accident or marked more than one answer.

Statistically significant differences were found in perceived safety competence scores according to department, willingness in department choice, health insurance status, adequacy of training, pre-practice screening, and awareness of safety measures. Fourth-year and midwifery students had the highest scores.

The mean score obtained from the Occupational Health and Safety Perceived Competency Scale was 103.06 ± 18.85 (min:43, max:145). Independent samples t-tests and analysis of variance (ANOVA) were conducted to compare participants’ socio-demographic and occupational health and safety-related characteristics with their mean scale scores. Mean scores differed significantly according to factors such as department choice, health insurance status, occupational health and safety training, pre-practice screening, and awareness of safety measures (p < .05). Tukey post hoc test results indicated that the significant difference was between second- and fourthyear students (p < .022), with fourth-year students obtaining the highest overall scale scores (Table 4).

**Table 4:** Distribution of the scores obtained from the occupational health and safety perceived competency scale according to some variables – Izmir, Türkiye, 2022.

Variables	N	Occupational health and safety perceived competency scal		
		X ± SD	t/F	*p*
**Department**				
Nursing	458	97.26 ± 17.45	11.474	**0.000**
Midwifery	289	112.25 ± 17.29		
**Choosing the department willingly**				
Yes	496	105.60 ± 18.74	5.271	**0.000**
No	251	98.03 ± 18.09		
**Year at school**				
2	254	101.04 ± 18.92	3.848	**0.022**
3	207	102.30 ± 18.96		
4	286	105.40 ± 18.52		
**Place of residence**				
Dormitory	414	103.36 ± 19.39	1.858	0.157
Home with the family	167	104.59 ± 18.77		
Home shared with friends	166	100.75 ± 17.40		
**Health insurance**				
Yes	594	104.21 ± 18.55	3.309	**0.001**
No	153	98.59 ± 19.40		
**Considering the occupational health and safety training received as adequate**				
Yes	570	105.91 ± 17.42	7.69	**0.000**
No	177	93.89 ± 20.37		
**Having a regular health screening before clinical practice**				
Yes	337	109.27 ± 17.74	8.545	**0.000**
No	410	97.95 ± 18.21		
**Having had an occupational accident**				
Yes	93	104.60 ± 18.39	.841	0.401
No	654	102.84 ± 18.92		
**Being aware of the measures to be taken to prevent occupational accidents**				
Yes	668	105.33 ± 17.50	10.213	**0.000**
No	79	83.86 ± 19.08		

The regression model indicated that department, voluntary choice of department, adequacy of training, and awareness of safety measures significantly predicted perceived safety competence, explaining 23.7% of the variance in scores.

The results of the multiple linear regression analysis examining the effects of the students’ descriptive variables on their occupational health and safety perceived competency are presented in Table 5. In the model, the variables explained 23.7% of the variance in perceived competency. According to the model, significant relationships were found between the mean scale score and variables such as department (p = .000), voluntary choice of department (p = .005), perceived adequacy of occupational health and safety training sufficient (p = .016), and awareness of occupational health and safety measures (p = .000).

**Table 5:** Analysis of factors affecting occupational health and safety perceived competency scale scores by multiple linear regression analysis – Izmir, Türkiye, 2022.

Variables	B	β	t	95.0% confidence interval	
				Lower limit	Upper limit	*p*
Constant term	113.097		108.551	111.052	115.142	.000
Department	–10.911	–.282	–8.174	–13.532	–8.291	.000
Choosing the department willingly	–3.684	–.092	–2.798	–6.269	–1.099	.005
Considering the occupational health and safety training received as adequate	–3.852	–.087	–2.420	–6.978	–.727	.016
Being aware of the measures to be taken to prevent occupational accidents	–14.847	–.242	–6.854	–19.100	–10.595	.000
R^2^: 23.7%, F: 46.012						

Students in the midwifery department had mean scale scores that were approximately 10 points higher than those of nursing students. Similarly, mean scores were approximately 3, 3, and 14 points higher among students who chose their department willingly, considered their occupational health and safety training sufficient, and were knowledgeable about occupational health and safety measures, respectively (Table 5).

## DISCUSSION

Enhancing nursing and midwifery students’ knowledge and awareness of occupational health and safety is essential to prevent exposure to physical and psychological harm. Therefore, the course content should be structured in accordance with occupational health and safety standards and tailored to students’ training needs. In Türkiye, according to the Occupational Health and Safety Law published in the Official Gazette (June 30, 2012)^(2)^, students studying in health-related fields are required to receive occupational health and safety training. In the present study, participants reported that occupational health and safety training was most frequently delivered within practical courses. However, Canbaş et al.^(9)^ reported that an analysis of nursing and midwifery curricula in a health school revealed the absence of a dedicated occupational health and safety course. Among the students in the present study, 50.4% stated that they received information on occupational health and safety within other courses, while 37.7% reported receiving such training at the institutions where they completed their internships. Similarly, Qaraman et al.^(13)^ found that students who took a precautions course during clinical training had significantly higher knowledge, attitude, and practice scores compared to those who did not receive the course.

Understanding students’ awareness of the accidents or risks they may encounter during clinical practice enables the identification of deficiencies and the planning of appropriate preventive initiatives. In a study conducted with health school students, 26.3% responded “yes” to the question “Do you know which occupational accidents you may be exposed to?”, and 22.5% answered “yes, always” to the question “Do you know what to do if you experience an occupational accident?”^(9)^. Similarly, in the study by Eyi and Eyi^(14)^, almost all participating students demonstrated low levels of knowledge and awareness regarding occupational health and safety. In contrast, in the present study, the majority of nursing and midwifery students reported being knowledgeable about preventive measures against occupational accidents, which may be attributed to the fact that both groups received occupational health and safety training before clinical practice.

Students may be exposed to a wide range of hazards during clinical practice, from sharps injuries to X-ray exposure. Awareness of the most common types of accidents can support both healthcare institutions and educational institutions in coordinating preventive strategies. In the study by Savcı et al.^(5)^, conducted with health department students, 69.8% reported experiencing at least one occupational accident during their internship. The most frequent incidents were sharps injuries, which occurred primarily in surgical and internal medicine clinics. Similarly, in a study conducted with midwifery and nursing students in Ethiopia, 37.7% experienced at least one needlestick injury, 9.3% reported slipping on wet floors, and 2.3% were exposed to electrical accidents^(4)^. In a retrospective study examining occupational accidents among student nurses in Australia, the most common incidents included sharps injuries, fainting while observing or assisting medical procedures, accidents occurring during patient handling or transfer, and workplace violence^(15)^. Additionally, several studies reported the prevalence of sharps injuries among students as 26.2% in Jordan, 46.6% in Uganda, 18.2% in Oman, 25% in India, and 16.8% in Türkiye^(16,17,18,19,20)^. Consistent with the existing literature, the present study found that students most frequently experienced sharps injuries, contamination with patients’ blood or body fluids, and falls. However, the frequency of these incidents was lower compared to some previous studies. This difference may be associated with the comprehensive occupational health and safety training provided to students, as well as their participation in occupational risk management and clinical orientation programs prior to clinical practice.

Reporting accidents and incidents is essential for improving occupational safety systems. Such reports can serve as a trigger for initiating improvement processes through the “plan– do–check–act” (PDCA) cycle^(21)^. In the study by Gök and Kaya^(15)^, 43.7% of student nurses experienced an occupational accident, 31% sustained sharps injuries, and 67.6% did not report the incident. Similarly, in the study by Suliman et al.^(20)^, many students did not know how to respond to needlestick injuries. Among these students, 67.1% did not inform their clinical instructors, and 86.3% failed to report the incident. In contrast, the proportion of students who did not report accidents was low in the present study. This finding may be attributed to the emphasis placed on incident reporting during training and clinical orientation programs. Additionally, students were informed that the reporting system was confidential, that reported information would not be used against them, and that feedback was provided regarding the root causes of reported incidents.

Students in health-related fields are expected to develop clinical skills through procedures that involve the use of sharp and piercing instruments, which may increase the risk of accidental exposure to bodily fluids. Some studies have reported that the risk of occupational accidents is higher among students than among healthcare employees. In addition to limited knowledge, skills, and experience in using clinical instruments, factors such as inattention, stress, sleep deprivation, and fatigue may increase students’ vulnerability to accidents and injuries^(22,23)^. In the study by Papadopoli et al.^(24)^, 49.1% of students reported inattention as the primary cause of accidents or injuries, while 31.6% attributed them to a lack of experience or practical skills. Similarly, another study found that injuries were attributed to the lack of personal protective equipment, crowded working environments, and insufficient procedural knowledge and experience by 24.2%, 17.3%, and 11.3% of nursing and midwifery students, respectively^(25)^.

The factors associated with accident exposure in the present study are consistent with those reported in the literature. Addressing these factors through appropriate preventive strategies may contribute to reducing the incidence of occupational accidents among students.

In recent years, the emphasis on developing safety awareness and fostering a culture of accident prevention at the international level has increased. Within this framework, occupational health and safety training represents a critical step in promoting safe practices. Such training enables individuals to translate theoretical knowledge into appropriate behavioral practices^(26)^. In the study by Juibari et al., the rate of occupational injuries decreased significantly as nurses’ knowledge of occupational health and safety increased^(27)^. Similarly, a study conducted with dentistry students reported a significant relationship between academic level and students’ knowledge and attitudes regarding occupational health and safety^(28)^. Consistent with these findings, the present study showed that fourth-year students obtained the highest scores on the Occupational Health and Safety Perceived Competency Scale, which may be attributed to their cumulative knowledge gained through clinical training and applied coursework over time. Midwifery students scored higher than nursing students, possibly because their curriculum includes a dedicated occupational health and safety course.

Regular health screenings are recommended for all healthcare professionals to help them cope with occupational hazards and to improve healthcare standards. In the study by Ojong et al.^(29)^, although healthcare professionals demonstrated high levels of knowledge regarding routine health screenings, the majority did not undergo such screenings. Similarly, in the study by Elewa and El Banan^(30)^, 88.0% of students reported that training programs on occupational hazards were insufficient, and 80.6% stated that they did not receive regular medical examinations. In Türkiye, pre-clinical health screening for students is optional and provided free of charge, and it is the students’ responsibility to schedule appointments. In the present study, students who underwent health screening before clinical practice obtained significantly higher scores on the Occupational Health and Safety Perceived Competency Scale compared to those who did not. This finding may reflect the positive influence of awareness gained through education on health-related behaviors.

Professional awareness is also important in occupational health and safety, as it is in many other issues. The study emphasizes that healthcare professionals must prioritize occupational health and safety throughout their careers. Multiple linear regression analysis showed that factors like department choice, willingness to choose the department, perception of sufficient safety training, and knowledge of safety measures significantly impact safety competence. It’s crucial to instill this awareness in students to ensure their own safety and that of their future patients.

### Study Limitations

This study has several limitations. First, the data were collected using self-reported questionnaires, which may be subject to recall bias and social desirability bias. Second, the study was conducted at only two universities located in the Aegean region of Türkiye, which may limit the generalizability of the findings to other regions or institutions. Finally, the cross-sectional design of the study prevents establishments of causal relationships between variables.

## CONCLUSION

This study demonstrated that both midwifery and nursing students are exposed to various occupational health hazards during clinical practice, with sharps injuries being the most frequently reported incident. Although most students received occupational health and safety training—primarily through applied courses—those who perceived the training as adequate, chose their departments willingly, and were aware of safety measures showed significantly higher levels of perceived safety competence. Additionally, midwifery students and fourth-year students achieved higher competency scores, which may be attributed to curriculum content and cumulative clinical experience.

These findings highlight the importance of integrating structured and standardized occupational health and safety training into the core curricula of health education programs. Moreover, fostering awareness through both theoretical and practical approaches may enhance students’ perceptions of safety competence. Educational background, motivation for professional choice, and awareness of preventive measures should be considered when designing effective training modules.

This study contributes to the literature by addressing a gap regarding occupational safety perceptions among healthcare students in Türkiye, particularly through its comparative analysis of nursing and midwifery students. Although several international studies have examined sharps injuries and safety training, relatively few have explored how student characteristics, educational context, and institutional awareness collectively influence perceived competency levels. In this regard, the study provides empirical evidence supporting the integration of comprehensive occupational health and safety education into undergraduate health curricula.

The findings also have practical implications for educational institutions, clinical educators, and policymakers. Developing tailored training modules, ensuring regular health screenings before clinical placements, and fostering a strong safety culture may help reduce occupational accidents and improve student preparedness. Furthermore, establishing a standardized national framework for occupational health and safety training across healthcare disciplines in Türkiye could help address current inconsistencies in training quality.

Overall, this study suggests that improving students’ perceptions of safety competence requires more than individual awareness; it necessitates coordinated efforts between academic institutions and clinical environments to protect future healthcare professionals and promote safe care settings.

## Data Availability

The entire dataset supporting the results of this study is available upon request to the corresponding author.
