# Supplementary material for: Midwifery and nursing students’ occupational health hazards and safety competence perceptions: a cross-sectional study from Türkiye
Source: Rev Esc Enferm USP. 2026 May 15;60:e20250085. doi: 10.1590/1980-220X-REEUSP-2025-0085en (PMC13179039; doi:10.1590/1980-220X-REEUSP-2025-0085en)
Supplement: Translation

# Translation: Riesgos para la salud ocupacional y percepciones de competencia en seguridad en estudiantes de obstetricia y enfermería: un estudio transversal en Turquía

**DOI:** 10.1590/1980-220X-REEUSP-2025-0085es

**Authors:** Aytül Hadımlı, Nurten Alan, İlkay Ünal, Nazan Tuna Oran

**Affiliations:** 1 Ege University, Faculty of Health Science, Department of Midwifery, Izmir,Türkiye.; 2 Dokuz Eylül University, Faculty of Nursing, Izmir, Türkiye.

**Keywords:** Salud Laboral, Enfermería, Partería, Estudiantes

## Abstract

**Objetivo:**

El estudio tuvo como objetivo investigar los riesgos para la salud ocupacional a los que están expuestos los estudiantes de obstetricia y enfermería durante su práctica clínica y sus percepciones de competencia en seguridad ocupacional.

**Método:**

El estudio incluyó a 289 estudiantes de obstetricia y 458 estudiantes de enfermería. Los datos se recopilaron mediante el Formulario de Información del Estudiante y la Escala de Competencia Percibida en Salud y Seguridad Ocupacional.

**Resultados:**

Durante la práctica clínica, los estudiantes estuvieron expuestos con mayor frecuencia a lesiones por objetos punzocortantes. La mayoría de los estudiantes que experimentaron un accidente ocupacional notificaron el incidente a más de una autoridad. El análisis de regresión linear múltipla indicó que los factores que predijeron las percepciones de competencia en seguridad ocupacional fueron la carrera cursada, haber elegido la carrera voluntariamente, percibir la formación como adecuada y estar informado sobre las medidas de salud y seguridad ocupacional.

**Conclusión:**

Este estudio identificó que los estudiantes de obstetricia y enfermería están expuestos con mayor frecuencia a lesiones por objetos punzocortantes durante la práctica clínica, principalmente debido a la inexperiencia y la falta de atención. Los estudiantes que recibieron formación adecuada en salud y seguridad ocupacional, eligieron su carrera voluntariamente y estaban informados sobre las medidas preventivas demostraron mayor competencia percibida en seguridad ocupacional. Los hallazgos confirman que tanto la formación educativa como los factores personales afectan significativamente las percepciones de seguridad de los estudiantes, lo que resalta la necesidad de una formación integral y estandarizada en salud y seguridad ocupacional en los programas de educación en salud.

## INTRODUCCIÓN

La Organización Internacional del Trabajo (OIT) define la salud y la seguridad ocupacional como la prevención de lesiones y enfermedades relacionadas con el trabajo y la protección y promoción de la salud de los trabajadores. También tiene como objetivo mejorar las condiciones de trabajo y el entorno laboral. La OIT informa que aproximadamente 2.3 millones de trabajadores en todo el mundo se ven afectados cada año por accidentes o enfermedades relacionados con el trabajo. En Turquía, a pesar de las precauciones tomadas, la prevalencia de los accidentes laborales sigue siendo alta. Según datos de la OIT de 2016, la tasa de lesiones laborales no mortales por cada 100 000 trabajadores en Turquía era de 1530, mientras que la tasa de mortalidad era de 7,5^(1)^. En Turquía, la Ley n.º 6331 ha regulado legalmente tanto las condiciones de salud y seguridad de los empleados como su formación en materia de salud y seguridad en el trabajo. Según el artículo 7/3 de esta ley, «es responsabilidad del empleador proporcionar formación y capacitación práctica tanto a los aprendices como a los pasantes». Sin embargo, los aprendices y los estudiantes que realizan prácticas en instituciones también deben recibir formación en materia de salud y seguridad en el trabajo. Según la ley, los cursos de salud y seguridad en el trabajo realizados por los aprendices y pasantes en sus instituciones educativas pueden sustituir a la formación básica en el lugar de trabajo si el empleador lo aprueba^(2)^.

Los empleados pueden enfrentarse a muchos peligros dependiendo de la naturaleza de su trabajo. El sector sanitario conlleva un mayor riesgo de accidentes y enfermedades laborales en comparación con muchos otros sectores. Los hospitales están clasificados como lugares de trabajo muy peligrosos según el «Comunicado sobre clases de peligros en el lugar de trabajo»^(3,4,5)^. Este sector, que supone un riesgo para los trabajadores sanitarios, también presenta un riesgo para los estudiantes de disciplinas sanitarias que se preparan para entrar en este campo^(6)^. Los estudios indican que muchos accidentes hospitalarios involucran a estudiantes, y aproximadamente la mitad sufre al menos un accidente durante las prácticas, siendo los más comunes las lesiones por objetos punzantes^(4,5,7,8,9,10,11)^. Los estudiantes de enfermería y partería, que constituyen uno de los grupos más numerosos en la educación sanitaria, se enfrentan a numerosos riesgos laborales durante la práctica clínica. Su limitada experiencia y conocimientos pueden aumentar el riesgo de accidentes laborales y enfermedades profesionales. Por lo tanto, es esencial dotarles de los conocimientos y la conciencia adecuados sobre salud y seguridad en el trabajo. El objetivo de este estudio es examinar los riesgos para la salud ocupacional a los que se enfrentan los estudiantes de partería y enfermería durante la práctica clínica y sus percepciones sobre la competencia en materia de seguridad ocupacional.

## MÉTODO

### Diseño del Estudio

Se trata de un estudio transversal.

### Definición de la Muestra

El estudio se llevó a cabo en dos universidades de la región del Egeo, en Turquía, entre octubre y diciembre de 2022. Estas universidades fueron seleccionadas porque establecieron programas de partería y enfermería con una gran población estudiantil y redes de prácticas clínicas accesibles. Se invitó a los estudiantes a participar al comienzo del semestre de otoño en octubre de 2022, y la recolección de datos continuó hasta diciembre de 2022.

Se incluyeron en el estudio estudiantes de enfermería de una de las universidades y estudiantes de partería de la otra universidad. Se consideró que todos los estudiantes de segundo, tercer y cuarto año que hubieran completado al menos un trimestre de prácticas clínicas eran aptos para participar.

Como parte de su plan de estudios de licenciatura, estos estudiantes ya habían recibido formación teórica formal sobre control de infecciones, seguridad de los pacientes y del lugar de trabajo, y procedimientos clínicos antes de comenzar sus prácticas. Los estudiantes que aún no habían comenzado las prácticas clínicas o que se negaron a dar su consentimiento informado fueron excluidos del estudio. Entre los estudiantes elegibles, 370 eran del Departamento de Partería y 790 del Departamento de Enfermería. De ellos, 289 estudiantes de partería y 458 de enfermería aceptaron participar. Por lo tanto, el estudio se llevó a cabo con 747 estudiantes. La selección se realizó mediante anuncios en clase por parte de los instructores e invitaciones por correo electrónico institucional. La participación fue voluntaria y se obtuvo el consentimiento informado por escrito de todos los participantes.

Las tasas de participación se calcularon por separado para cada departamento. En el Departamento de Partería, participaron 289 de los 370 estudiantes elegibles, lo que supuso una tasa de participación del 78,1 %. En el Departamento de Enfermería, participaron 458 de los 790 estudiantes elegibles, lo que supuso una tasa de participación del 58,0 %.

La adecuación del tamaño de la muestra de 747 estudiantes se evaluó mediante un análisis de potencia post hoc basado en un nivel de confianza del 95 %, un nivel de significación del 5 % (α = 0,05) y un tamaño del efecto medio (d de Cohen = 0,5). El análisis indicó que la potencia estadística del estudio superaba el 95 %, lo que demostraba que el tamaño de la muestra era suficiente para los análisis estadísticos.

### Recolección de Datos

*Formulario de información del estudiante:* El formulario, elaborado de acuerdo con la bibliografía, consta de 33 ítems^(5,9)^. De estos ítems, 12 evalúan las características sociodemográficas de los estudiantes y 11 evalúan sus conocimientos y experiencia relacionados con la salud y la seguridad en el trabajo. El formulario de información del estudiante consta de dos secciones: (a) 12 preguntas sobre características sociodemográficas (por ejemplo, edad, género, departamento, año de estudio, nivel de ingresos y enfermedades crónicas); y (b) 11 preguntas que evalúan los conocimientos y la experiencia relacionados con la salud y la seguridad en el trabajo, incluyendo la formación previa, el conocimiento de las medidas de protección, el historial de accidentes laborales y el comportamiento de notificación.

*Escala de competencia percibida en materia de salud y seguridad en el trabajo (Occupational Health and Safety Perceived Competency Scale)*: La escala, desarrollada por Kocaay y Ocaktan para evaluar la percepción de los empleados sobre la competencia en materia de salud y seguridad en el trabajo, consta de 29 ítems. Las respuestas a los ítems se califican en una escala tipo Likert de cinco puntos que va del 1 al 5 (1 = nada, 2 = un poco, 3 = en parte, 4 = bien y 5 = muy bien). Ningún ítem se puntúa de forma inversa. Las puntuaciones mínimas y máximas que se pueden obtener en la escala son 29 y 145, respectivamente. El coeficiente de fiabilidad alfa de Cronbach de la escala fue de 0,91 en el estudio original de Kocaay y Ocaktan^(12)^, y de 0,97 en el presente estudio, lo que indica una excelente consistencia interna.

### Análisis de Datos

Los datos obtenidos del estudio se analizaron utilizando el programa SPSS 25.0. La normalidad de la distribución de los datos se evaluó mediante la prueba de Kolmogorov-Smirnov. Dado que los datos se distribuían normalmente, se aplicaron pruebas paramétricas.

Para comparar las puntuaciones medias de la Escala de Competencia Percibida en Salud y Seguridad Ocupacional entre variables independientes, se utilizó la prueba t para muestras independientes para las comparaciones entre dos grupos, mientras que se utilizó el análisis de varianza unidireccional (ANOVA), el análisis post hoc HSD de Tukey y el análisis de regresión lineal múltiple para las comparaciones entre más de dos grupos. Se consideró estadísticamente significativo un p-valor inferior a 0,05.

### Aspectos Éticos

Este estudio fue aprobado por el Comité de Ética en Investigación Científica y Publicaciones de la Universidad XXX (Protocolo n.º: 09/12-1580). Se informó a los estudiantes participantes sobre el propósito y el alcance del estudio, y se obtuvo el consentimiento informado por escrito de todos los participantes.

## RESULTADOS

La edad media de los estudiantes que participaron en el estudio fue de 21,10 ± 1,50 (mínimo: 19, máximo: 33) años. El 76,6 % de los participantes eran mujeres, el 61,3 % eran estudiantes de enfermería y el 38,7 % eran estudiantes de partería. Además, el 55,4 % vivía en residencias, el 53,1 % declaró tener unos ingresos equivalentes a sus gastos, el 90,9 % no tenía un trabajo remunerado, el 79,5 % tenía seguro médico y el 93,0 % no padecía ninguna enfermedad crónica (Tabla 1).

**Tabla 1:** Características sociodemográficas y relacionadas con la salud de los estudiantes participantes – Esmirna, Turquía, 2022.

Características	Número	%
**Edad media:** 21,10 ± 1,50 (mín.: 19, máx.: 33) años		
**Sexo**		
Mujer	572	76,6
Hombres	175	23,4
**Departamento**		
Enfermería	458	61,3
Partería	289	38,7
**Años de escolaridad**		
2	254	34,0
3	207	27,7
4	286	38,3
**Lugar de residencia**		
Casa compartida con amigos	166	22,2
Hogar con la familia	167	22,4
Residencia	414	55,4
**Nivel de ingresos**		
Ingresos superiores a los gastos	85	11,4
Ingresos iguales a los gastos	397	53,1
Ingresos inferiores a los gastos	265	35,5
**Situación laboral**		
Empleado	68	9,1
Sin empleo	679	90,9
**Seguro médico**		
Sí	594	79,5
No	153	20,5
**Presencia de una enfermedad por la que recibe tratamiento constante**		
Sí	52	7,0
No	695	93,0
**Tipo de enfermedad (n: 52)**		
Sistema endocrino	14	26,9
Sistema inmunitario	9	17,3
Sistema gastrointestinal	8	15,4
Sistema nervioso	6	11,5
Sistema cardiovascular	5	9,6
Sistema respiratorio	5	9,6
Psiquiátrico	5	9,6
**Total**	**747**	**100**

La mayoría de los participantes eran estudiantes de enfermería de segundo o cuarto año, la mayoría de las cuales residían en residencias universitarias. La mayoría tenía seguro médico, no trabajaba y no padecía ninguna enfermedad crónica.

La mayoría de los estudiantes afirmaron haber recibido formación en materia de salud y seguridad en el trabajo en el marco de cursos aplicados y consideraron que dicha formación era adecuada. Durante la formación clínica se practicaron con frecuencia medidas de protección básicas, como la higiene de las manos, el uso de guantes y el uso de mascarillas.

Los estudiantes recibieron formación en materia de salud y seguridad en el trabajo en diversos entornos, sobre todo como parte de cursos prácticos. Un total del 76,3 % afirmó que la formación recibida era suficiente. Durante la práctica clínica, las medidas de protección más utilizadas fueron la higiene de las manos, los guantes y las mascarillas (Tabla 2).

**Tabla 2:** Resultados relativos a la educación en materia de salud y seguridad en el trabajo de los estudiantes – Esmirna, Turquía, 2022.

Características	Número	%
**Lugar donde reciben formación en salud laboral^†^**
Curso de salud y seguridad laboral en la escuela	295	39,5
Dentro del ámbito de los cursos aplicados	501	67,1
En la institución donde el participante realiza sus prácticas	54	7,2
En un curso privado que imparte formación en salud laboral	79	10,6
**Considera que la formación recibida en materia de salud y seguridad en el trabajo es adecuada**
Sí	570	76,3
No	177	23,7
**Conocer las medidas que se deben tomar para prevenir accidentes laborales**
Sí	668	89,4
No	79	10,6
**Realizar revisiones médicas periódicas antes de la práctica clínica**
Sí	337	45,1
No	410	54,9
**Medidas de protección adoptadas durante la práctica clínica^†^**
Ninguna	7	0,9
Lavado de manos	684	91,6
Uso de guantes	675	90,4
Usar mascarilla	645	86,3
Uso de gafas/viseras	41	5,5
Uso de delantal	278	37,2
**Total**	**747**	**100**

^†^Respuestas multiples.

El accidente laboral más común fueron las lesiones por objetos punzantes, causadas principalmente por falta de atención o falta de experiencia. La mayoría de los incidentes se notificaron, normalmente a la enfermera formadora o a las unidades institucionales pertinentes.

Entre los estudiantes, el 87,6 % afirmó no haber sufrido ningún accidente laboral durante sus prácticas clínicas. El tipo de accidente más común fueron las lesiones por objetos punzantes, y la causa más frecuente fue el descuido. Los estudiantes que sufrieron un accidente laboral informaron del incidente a más de una autoridad. La enfermera formadora fue la autoridad a la que se informaron más incidentes (Tabla 3).

**Tabla 3:** Resultados sobre los accidentes laborales que sufrieron los estudiantes participantes – Esmirna, Turquía, 2022.

Características	Número	%
**Haber sufrido un accidente laboral**		
Sí	93	12,4
No	654	87,6
**Tipo de accidente laboral^‡^**		
Lesiones por objetos punzantes	77	10,8
Contaminación con sangre o fluidos corporales del paciente	14	1,9
Caídas	9	1,2
Exposición a violencia física/verbal	4	0,5
Exposición a rayos X	1	0,1
Lesiones químicas	1	0,1
**Causa del accidente^‡^**		
Desatención	59	7,9
Falta de práctica	33	4,4
Falta de equipo de protección personal	13	1,7
Fatiga, insomnio	17	2,3
Estrés, ansiedad	12	1,6
**Autoridad a la que se notifica el accidente^‡^**		
La enfermera que imparte la formación	37	5,0
Unidad de salud y seguridad de los empleados del hospital	32	4,3
Profesor/supervisor de solicitudes de prácticas	30	4,0
Unidad de control de infecciones	17	2,3
Sin notificación	28	3,7

^‡^Estudiantes que sufrieron un accidente o marcaron más de una respuesta.

Se encontraron diferencias estadísticamente significativas en las puntuaciones de competencia en materia de seguridad percibida según el departamento, la disposición en la elección del departamento, la situación del seguro médico, la adecuación de la formación, la selección previa a la práctica y el conocimiento de las medidas de seguridad. Los estudiantes de cuarto año y de obstetricia obtuvieron las puntuaciones más altas.

La puntuación media obtenida en la Escala de Competencia Percibida en Salud y Seguridad Ocupacional fue de 103,06 ± 18,85 (mínimo: 43, máximo: 145). Se realizaron pruebas t para muestras independientes y análisis de varianza (ANOVA) para comparar las características sociodemográficas y relacionadas con la salud y la seguridad ocupacional de los participantes con sus puntuaciones medias en la escala. Las puntuaciones medias difirieron significativamente en función de factores como la elección del departamento, la situación en materia de seguro médico, la formación en salud y seguridad en el trabajo, la evaluación previa a la práctica y el conocimiento de las medidas de seguridad (p < 0,05). Los resultados de la prueba post hoc de Tukey indicaron que la diferencia significativa se daba entre los estudiantes de segundo y cuarto año (p < 0,022), siendo los estudiantes de cuarto año los que obtuvieron las puntuaciones globales más altas en la escala (Tabla 4).

**Tabla 4:** Distribución de las puntuaciones obtenidas en la escala de competencia percibida en materia de salud y seguridad en el trabajo según algunas variables – Esmirna, Turquía, 2022.

Variables	N	Escala de competencia percibida en materia de salud y seguridad en el trabajo		
		X ± DE	t/F	*p*
**Departamento**				
Enfermería	458	97,26 ± 17,45	11,474	**0,000**
Partería	289	112,25 ± 17,29		
**Elección voluntaria del departamento**				
Sí	496	105,60 ± 18,74	5,271	**0,000**
Mo	251	98,03 ± 18,09		
**Año escolar**				
2	254	101,04 ± 18,92	3,848	**0,022**
3	207	102,30 ± 18,96		
4	286	105,40 ± 18,52		
**Lugar de residencia**				
Residencia	414	103,36 ± 19,39	1,858	0,157
Hogar con la familia	167	104,59 ± 18,77		
Hogar compartido con amigos	166	100,75 ± 17,40		
**Seguro médico**				
Sí	594	104,21 ± 18,55	3,309	**0,001**
No	153	98,59 ± 19,40		
**Considera que la formación recibida en materia de salud y seguridad en el trabajo es adecuada**				
Sí	570	105,91 ± 17,42	7,69	**0,000**
No	177	93,89 ± 20,37		
**Realiza revisiones médicas periódicas antes de la práctica clínica**				
Sí	337	109,27 ± 17,74	8,545	**0,000**
No	410	97,95 ± 18,21		
**Ha sufrido un accidente laboral**				
Sí	93	104,60 ± 18,39	0,841	0,401
No	654	102,84 ± 18,92		
**Conocimiento de las medidas que deben adoptarse para prevenir los accidentes laborales**				
Sí	668	105,33 ± 17,50	10,213	**0,000**
No	79	83,86 ± 19,08		

El modelo de regresión indicó que el departamento, la elección voluntaria del departamento, la adecuación de la formación y el conocimiento de las medidas de seguridad predecían de manera significativa la competencia percibida en materia de seguridad, lo que explicaba el 23,7 % de la varianza en las puntuaciones.

Los resultados del análisis de regresión lineal múltiple que examina los efectos de las variables descriptivas de los estudiantes sobre su competencia percibida en materia de salud y seguridad en el trabajo se presentan en la Tabla 5. En el modelo, las variables explicaban el 23,7 % de la varianza en la competencia percibida. Según el modelo, se encontraron relaciones significativas entre la puntuación media de la escala y variables como el departamento (p = 0,000), la elección voluntaria del departamento (p = 0,005), la percepción de que la formación en salud y seguridad ocupacional era suficiente (p = 0,016) y el conocimiento de las medidas de salud y seguridad ocupacional (p = 0,000).

**Tabla 5:** Análisis de los factores que afectan a las puntuaciones de la escala de competencia percibida en materia de salud y seguridad en el trabajo mediante análisis de regresión lineal múltiple – Esmirna, Turquía, 2022.

Variables	B	β	t	Intervalo de confianza del 95,0 %		*p*
				Límite inferior	Límite superior	
Término constante	113,097		108,551	111,052	115,142	.000
Departamento	–10,911	–0,282	–8,174	–13,532	–8,291	.000
Elegir el departamento voluntariamente	–3,684	–0,092	–2,798	–6,269	–1,099	0,005
Considerando que la formación en materia de salud y seguridad en el trabajo recibida es adecuada	–3,852	–0,087	–2,420	–6,978	–,727	0,016
Conocer las medidas que se deben tomar para prevenir los accidentes laborales	–14,847	–0,242	–6,854	–19,100	–10,595	.000
R^2^: 23,7%, F: 46,012						

Los estudiantes del departamento de partería obtuvieron puntuaciones medias en la escala aproximadamente 10 puntos más altas que las de los estudiantes de enfermería. Del mismo modo, las puntuaciones medias fueron aproximadamente 3, 3 y 14 puntos más altas entre los estudiantes que eligieron su departamento voluntariamente, consideraron que su formación en salud y seguridad en el trabajo era suficiente y conocían las medidas de salud y seguridad en el trabajo, respectivamente (Tabla 5).

## DISCUSIÓN

Mejorar los conocimientos y la concienciación de los estudiantes de enfermería y partería sobre la salud y la seguridad en el trabajo es esencial para prevenir la exposición a daños físicos y psicológicos. Por lo tanto, el contenido del curso debe estructurarse de acuerdo con las normas de salud y seguridad en el trabajo y adaptarse a las necesidades de formación de los estudiantes. En Turquía, de acuerdo con la Ley de Salud y Seguridad en el Trabajo publicada en el Boletín Oficial (30 de junio de 2012)^(2)^, los estudiantes de campos relacionados con la salud deben recibir formación en materia de salud y seguridad en el trabajo. En el presente estudio, los participantes informaron de que la formación en materia de salud y seguridad en el trabajo se impartía con mayor frecuencia en los cursos prácticos. Sin embargo, Canbaş et al.^(9)^ informaron de que un análisis de los planes de estudios de enfermería y partería en una escuela de salud reveló la ausencia de un curso específico sobre salud y seguridad en el trabajo. Entre los estudiantes del presente estudio, el 50,4 % afirmó haber recibido información sobre salud y seguridad en el trabajo en otros cursos, mientras que el 37,7 % informó haber recibido dicha formación en las instituciones donde realizaron sus prácticas. De manera similar, Qaraman et al.^(13)^ descubrieron que los estudiantes que tomaron un curso de precauciones durante la formación clínica tenían puntuaciones significativamente más altas en conocimientos, actitud y práctica en comparación con los que no recibieron el curso.

Comprender la conciencia de los estudiantes sobre los accidentes o riesgos a los que pueden enfrentarse durante la práctica clínica permite identificar las deficiencias y planificar iniciativas preventivas adecuadas. En un estudio realizado con estudiantes de escuelas de salud, el 26.3 % respondió «sí» a la pregunta «¿Sabes a qué accidentes laborales puedes estar expuesto?», y el 22.5 % respondió «sí, siempre» a la pregunta «¿Sabes qué hacer si sufres un accidente laboral?»^(9)^. Del mismo modo, en el estudio de Eyi y Eyi^(14)^, casi todos los estudiantes participantes demostraron bajos niveles de conocimiento y concienciación sobre la salud y la seguridad en el trabajo. Por el contrario, en el presente estudio, la mayoría de los estudiantes de enfermería y partería afirmaron tener conocimientos sobre las medidas preventivas contra los accidentes laborales, lo que puede atribuirse al hecho de que ambos grupos recibieron formación en materia de salud y seguridad en el trabajo antes de la práctica clínica.

Los estudiantes pueden estar expuestos a una amplia gama de riesgos durante la práctica clínica, desde lesiones por objetos punzantes hasta la exposición a rayos X. El conocimiento de los tipos de accidentes más comunes puede ayudar tanto a las instituciones sanitarias como a las educativas a coordinar estrategias preventivas. En el estudio de Savcı et al.^(5)^, realizado con estudiantes del departamento de salud, el 69.8 % informó haber sufrido al menos un accidente laboral durante sus prácticas. Los incidentes más frecuentes fueron las lesiones por objetos punzantes, que se produjeron principalmente en clínicas quirúrgicas y de medicina interna. Del mismo modo, en un estudio realizado con estudiantes de partería y enfermería en Etiopía, el 37,7 % sufrió al menos una lesión por pinchazo con aguja, el 9,3 % informó haber resbalado en suelos mojados y el 2,3 % estuvo expuesto a accidentes eléctricos^(4)^. En un estudio retrospectivo que examinó los accidentes laborales entre estudiantes de enfermería en Australia, los incidentes más comunes incluyeron lesiones por objetos punzantes, desmayos mientras observaban o asistían en procedimientos médicos, accidentes ocurridos durante la manipulación o el traslado de pacientes y violencia en el lugar de trabajo^(15)^. Además, varios estudios informaron de una prevalencia de lesiones por objetos punzantes entre los estudiantes del 26,2 % en Jordania, el 46,6 % en Uganda, el 18,2 % en Omán, el 25 % en la India y el 16,8 % en Turquía^(16,17,18,19,20)^. En consonancia con la bibliografía existente, el presente estudio reveló que los estudiantes sufrían con mayor frecuencia lesiones por objetos punzantes, contaminación con sangre o fluidos corporales de los pacientes y caídas. Sin embargo, la frecuencia de estos incidentes fue menor en comparación con algunos estudios anteriores. Esta diferencia puede estar relacionada con la formación integral en materia de salud y seguridad en el trabajo que se imparte a los estudiantes, así como con su participación en programas de gestión de riesgos laborales y orientación clínica antes de la práctica clínica.

Notificar los accidentes e incidentes es esencial para mejorar los sistemas de seguridad en el trabajo. Estos informes pueden servir de detonante para iniciar procesos de mejora a través del ciclo «planificar-hacer-verificar-actuar» (Plan-Do-Check-Act - PDCA)^(21)^. En el estudio de Gök y Kaya^(15)^, el 43.7 % de los estudiantes de enfermería sufrieron un accidente laboral, el 31 % sufrieron lesiones por objetos punzantes y el 67.6 % no informaron del incidente. Del mismo modo, en el estudio de Suliman et al.^(20)^, muchos estudiantes no sabían cómo responder a las lesiones por pinchazos con agujas. Entre estos estudiantes, el 67,1 % no informó a sus instructores clínicos y el 86,3 % no informó del incidente. Por el contrario, la proporción de estudiantes que no informaron de los accidentes fue baja en el presente estudio. Este hallazgo puede atribuirse al énfasis que se pone en la notificación de incidentes durante los programas de formación y orientación clínica. Además, se informó a los estudiantes de que el sistema de notificación era confidencial, que la información notificada no se utilizaría en su contra y que se les proporcionaría información sobre las causas fundamentales de los incidentes notificados.

Se espera que los estudiantes de campos relacionados con la salud desarrollen habilidades clínicas a través de procedimientos que implican el uso de instrumentos afilados y punzantes, lo que puede aumentar el riesgo de exposición accidental a fluidos corporales. Algunos estudios han informado de que el riesgo de accidentes laborales es mayor entre los estudiantes que entre los empleados del sector sanitario. Además de los conocimientos, las habilidades y la experiencia limitados en el uso de instrumentos clínicos, factores como la falta de atención, el estrés, la falta de sueño y la fatiga pueden aumentar la vulnerabilidad de los estudiantes a los accidentes y las lesiones^(22,23)^. En el estudio de Papadopoli et al.^(24)^, el 49,1 % de los estudiantes señalaron la falta de atención como la causa principal de los accidentes o lesiones, mientras que el 31,6 % los atribuyeron a la falta de experiencia o de habilidades prácticas. De manera similar, otro estudio reveló que las lesiones se atribuían a la falta de equipo de protección personal, a los entornos de trabajo abarrotados y al conocimiento y la experiencia insuficientes en materia de procedimientos por parte del 24,2 %, el 17,3 % y el 11,3 % de los estudiantes de enfermería y partería, respectivamente^(25)^.

Los factores asociados con la exposición a accidentes en el presente estudio son coherentes con los descritos en la literatura. Abordar estos factores mediante estrategias preventivas adecuadas puede contribuir a reducir la incidencia de accidentes laborales entre los estudiantes.

En los últimos años, ha aumentado el énfasis en el desarrollo de la conciencia sobre la seguridad y el fomento de una cultura de prevención de accidentes a nivel internacional. En este marco, la formación en materia de salud y seguridad en el trabajo representa un paso fundamental para promover prácticas seguras. Esta formación permite a las personas traducir los conocimientos teóricos en prácticas de comportamiento adecuadas^(26)^. En el estudio de Juibari et al., la tasa de lesiones laborales disminuyó significativamente a medida que aumentaban los conocimientos de las enfermeras sobre salud y seguridad en el trabajo^(27)^. Del mismo modo, un estudio realizado con estudiantes de odontología reveló una relación significativa entre el nivel académico y los conocimientos y actitudes de los estudiantes en materia de salud y seguridad en el trabajo^(28)^. En consonancia con estos hallazgos, el presente estudio mostró que los estudiantes de cuarto año obtuvieron las puntuaciones más altas en la Escala de Competencia Percibida en Salud y Seguridad Ocupacional, lo que puede atribuirse a los conocimientos acumulados a lo largo del tiempo a través de la formación clínica y los cursos aplicados. Los estudiantes de partería obtuvieron puntuaciones más altas que los de enfermería, posiblemente porque su plan de estudios incluye un curso específico sobre salud y seguridad ocupacional.

Se recomienda que todos los profesionales de la salud se sometan a exámenes médicos periódicos para ayudarles a hacer frente a los riesgos laborales y mejorar los estándares de atención médica. En el estudio de Ojong et al.^(29)^, aunque los profesionales de la salud demostraron un alto nivel de conocimiento sobre los exámenes médicos rutinarios, la mayoría no se sometió a dichos exámenes. Del mismo modo, en el estudio de Elewa y and El Banan^(30)^, el 88,0 % de los estudiantes informaron de que los programas de formación sobre riesgos laborales eran insuficientes, y el 80,6 % afirmaron que no se sometían a exámenes médicos periódicos. En Turquía, los exámenes médicos preclínicos para los estudiantes son opcionales y gratuitos, y es responsabilidad de los estudiantes programar las citas. En el presente estudio, los estudiantes que se sometieron a exámenes médicos antes de la práctica clínica obtuvieron puntuaciones significativamente más altas en la Escala de Competencia Percibida en Salud y Seguridad Ocupacional en comparación con los que no lo hicieron. Este hallazgo puede reflejar la influencia positiva de la concienciación adquirida a través de la educación sobre los comportamientos relacionados con la salud.

La conciencia profesional también es importante en la salud y la seguridad en el trabajo, al igual que en muchas otras cuestiones. El estudio hace hincapié en que los profesionales de la salud deben dar prioridad a la salud y la seguridad en el trabajo a lo largo de sus carreras. El análisis de regresión lineal múltiple mostró que factores como la elección del departamento, la disposición a elegir el departamento, la percepción de una formación suficiente en materia de seguridad y el conocimiento de las medidas de seguridad influyen significativamente en la competencia en materia de seguridad. Es fundamental inculcar esta conciencia en los estudiantes para garantizar su propia seguridad y la de sus futuros pacientes.

### Limitaciones del Estudio

Este estudio tiene varias limitaciones. En primer lugar, los datos se recolectaron mediante cuestionarios autoadministrados, que pueden estar sujetos a sesgos de recuerdo y de deseabilidad social. En segundo lugar, el estudio se llevó a cabo únicamente en dos universidades situadas en la región del Egeo de Turquía, lo que puede limitar la generalización de los resultados a otras regiones o instituciones. Por último, el diseño transversal del estudio impide establecer relaciones causales entre las variables.

## CONCLUSIÓN

Este estudio demostró que tanto los estudiantes de partería como los de enfermería están expuestos a diversos riesgos para la salud ocupacional durante la práctica clínica, siendo las lesiones por objetos punzantes el incidente más frecuente. Aunque la mayoría de los estudiantes recibieron formación en salud y seguridad ocupacional, principalmente a través de cursos aplicados, aquellos que percibieron la formación como adecuada, eligieron sus departamentos voluntariamente y eran conscientes de las medidas de seguridad mostraron niveles significativamente más altos de competencia percibida en materia de seguridad. Además, los estudiantes de partería y los de cuarto año obtuvieron puntuaciones de competencia más altas, lo que puede atribuirse al contenido del plan de estudios y a la experiencia clínica acumulada.

Estos hallazgos ponen de relieve la importancia de integrar una formación estructurada y estandarizada en materia de salud y seguridad ocupacional en los planes de estudios básicos de los programas de educación sanitaria. Además, fomentar la concienciación mediante enfoques tanto teóricos como prácticos puede mejorar la percepción de los estudiantes sobre la competencia en materia de seguridad. A la hora de diseñar módulos de formación eficaces, se deben tener en cuenta la formación académica, la motivación para la elección profesional y la concienciación sobre las medidas preventivas.

Este estudio contribuye a la literatura al abordar una laguna en relación con las percepciones de seguridad laboral entre los estudiantes de ciencias de la salud en Turquía, en particular a través de su análisis comparativo de los estudiantes de enfermería y partería. Aunque varios estudios internacionales han examinado las lesiones por objetos punzantes y la formación en materia de seguridad, son relativamente pocos los que han explorado cómo las características de los estudiantes, el contexto educativo y la conciencia institucional influyen colectivamente en los niveles de competencia percibidos. En este sentido, el estudio proporciona pruebas empíricas que respaldan la integración de una educación integral en materia de salud y seguridad laboral en los planes de estudios de grado en ciencias de la salud.

Los resultados también tienen implicaciones prácticas para las instituciones educativas, los educadores clínicos y los responsables políticos. El desarrollo de módulos de formación adaptados, la garantía de exámenes médicos periódicos antes de las prácticas clínicas y el fomento de una sólida cultura de seguridad pueden ayudar a reducir los accidentes laborales y mejorar la preparación de los estudiantes. Además, el establecimiento de un marco nacional estandarizado para la formación en salud y seguridad laboral en todas las disciplinas sanitarias en Turquía podría ayudar a abordar las inconsistencias actuales en la calidad de la formación.

En general, este estudio sugiere que mejorar la percepción de los estudiantes sobre la competencia en materia de seguridad requiere algo más que la concienciación individual; es necesario un esfuerzo coordinado entre las instituciones académicas y los entornos clínicos para proteger a los futuros profesionales de la salud y promover entornos de atención seguros.

## Data Availability

El conjunto de datos que respaldan las conclusiones de este estudio está disponible previa solicitud al autor correspondiente.
